# Ankle joint rotation and exerted moment during plantarflexion dependents on measuring- and fixation method

**DOI:** 10.1371/journal.pone.0253015

**Published:** 2021-08-31

**Authors:** Savvas Stafilidis, Carina Kopper-Zisser

**Affiliations:** Department of Biomechanics, Kinesiology and Computer Science in Sport, Institute of Sport Science, Sport, University of Vienna, Vienna, Austria; Universita degli Studi di Milano, ITALY

## Abstract

We examined the effect of ankle joint fixation vs increased foot pressure (aiming to reduce dynamometer-subject elasticity (DSE)) on the exerted moment during plantarflexion contraction. We also examined the joint rotation in dependence of the measuring site (forefoot, rearfoot) and the foot condition (fixed, free). We hypothesized higher exerted moments due to reduced DSE compared to fixed condition and an effect of fixation on the joint rotation in dependence of the measuring site. Fourteen healthy individuals (28.7±6.9y) completed in randomized order maximal isometric plantarflexions in four different positions (0-3-6-9 cm) and two ankle joint conditions (fixed-free). Kinematics of the rear- and forefoot were obtained synchronously. We found higher moment in the fixed compared to the free condition at all positions. The maximum moment in the fixed condition did not differ at any position. At the fixed condition, the forefoot rotation did not differ at any position (~5°) while at free condition we observed a significant rotation reduction (form ~12 to ~5°). The rearfoot rotation did not differ between conditions at any position while a significant joint angle reduction was observed (~10 to ~6° and ~12 to ~6°; fixed-free respectively). The results indicate that with appropriate foot fixation the maximum moment can be achieved irrespective of the position. With the foot secured, the measuring site influences the rotational outcome. We suggest that for a minimization of the joint rotation a fixation and the forefoot-measuring site should be preferred. Additionally, for unconstrained foot kinematic observations both measuring sites can be obtained.

## Introduction

The assessment of the mechano-morphological properties of the lower leg muscle-tendon unit was subject to numerous studies in the past [1–4]. Depending on the research question, different scientific measuring devices were used. For example for the assessment of the dynamic properties, custom- made [2,3,5–9] or commercially available isokinetic dynamometers [10–13] were implemented. To reduce the ankle joint movement, commercial dynamometer manufactures are suggesting securing the foot on the dynamometer footplate with inextensible straps. The majority of the conducted research [5–7,13–15] used the proposed fixation method but nonetheless other researchers [2,3,8,9,12,16–19] avoided to implement that procedure. A possible explanation could be the reliance on the increased rigidity of the custom-made dynamometer device or the scope of the research project.

It is known from the literature that during the plantarflexion efforts, an inevitable joint rotation occurs [1,12,20]. The origin of the joint rotation was identified on the compliance of the dynamometer-subject system [21], the cushioning pads [22] and the soft muscular tissue [23]. The joint rotation could have further implications on the assessment of the mechanical and morphological properties of the lower leg muscle tendon unit, since the muscle fibers would not operate at the desired length [20] or the tendon elongation could be overestimated [24]. For that purpose, post-processing correction methods were suggested that could solve the aforementioned drawbacks [23]. Moreover, other researchers implemented an alternative mechanical strategy to account for the compliance of the dynamometer-subject system [25,26]. In their attempt to reduce the ankle joint rotation the researchers repositioned the dynamometer chair prior to measurement forward and thus a 20–30° knee joint angle was developed. With the subsequent straightening of the knee joint, the cushioning pads were compressed and a firmer contact of the foot to the dynamometer plate was ensured. In a recent attempt [12] we showed that with a similar method, a foot pressure of ~220kPa was necessary in order to achieve the maximal plantarflexion moment and to reduce the ankle joint rotation (>32% and >50% respectively), compared to initial condition. That outcome raised the question, if the use of a fixation method (straps) is necessary to achieve the maximum moment and minimum joint rotation during plantar flexion efforts or if only chair adjustments is needed [12,25,26] to reach similar results.

Nonetheless, to assess the kinematics of the ankle joint during plantarflexion efforts, researchers implemented digital [1,26], infrared cameras [12,25,27], electrical goniometers [3,5,13], potentiometers [4] or simply measured the crank angle provided by the isokinetic dynamometer [28,29]. For example, Magnusson and colleagues (2001) [3] measured the joint rotation during plantarflexion efforts by placing an electrical goniometer on the distal part of the fifth metatarsal and the posterolateral aspect of the fibula. However, in order to capture the ankle joint kinematics with digital and infrared cameras it is necessary to use reflective markers. The positioning of the markers varies between studies depending on the capturing mode (2D-3D) and scope of the project. For example, Theis and colleagues (2012) [25] placed two reflective markers on the footplate and on the calcaneus and the distal end of the first metatarsal. The authors defined the angle change between the footplate and the foot as the ankle joint angle change. Similarly, other researchers [26,30] used the aforementioned method to monitor (2D) the heel rise during plantar flexion efforts. In another study, Muramatsu and colleagues (2001) [1] measured the ankle joint angle defined by reflective markers placed on the lateral epicondyle of femur, lateral malleolus and calcaneal tuber. Accordingly, we previously monitored the ankle joint rotation in the sagittal plane by placing five reflective markers on medial-lateral epicondyles and malleolus, and calcaneal tuber [12]. Nonetheless, it is known that the human foot is multi-articulated [31] and can be roughly divided in three (forefoot, midfoot, rearfoot) segments [32]. It appears that in the sagittal plane the segments have different kinematic characteristics independent of the movement task. For example, Arampatzis and colleagues (2002) showed that, during landings, the dorsiflexion angle was different between the forefoot and the rearfoot segment [33]. Also in a recent study [34] that examined gender differences in the rear-, mid- and forefoot angles during running, the authors showed greater dorsiflexion angles in the rearfoot than in the forefoot. Nonetheless, the different techniques and marker setups used to estimate the joint rotation appear to monitor either the forefoot or rearfoot and therefore it is reasonable to assume that also during isometric plantarflexion contractions the measuring site (forefoot-rearfoot) would affect the estimation of the ankle joint rotation.

Consequently, the aim of this study was to examine the effectiveness of the foot straps in comparison to the forward positioning method [12,25,26] when assessing the maximum achievable plantarflexion moment. Furthermore, we aimed to examine the difference of joint rotation when implementing two different measuring sites (forefoot-rearfoot). Based on previous findings, we hypothesized that the maximum exerted joint moment developed by forward positioning of the subject would be comparable with the moment produced when only the foot is securely fixed with straps. We also hypothesized that the implementation of different marker sets (forefoot-rearfoot) will result to different joint rotation estimations.

## Methods

**Fig 1:**
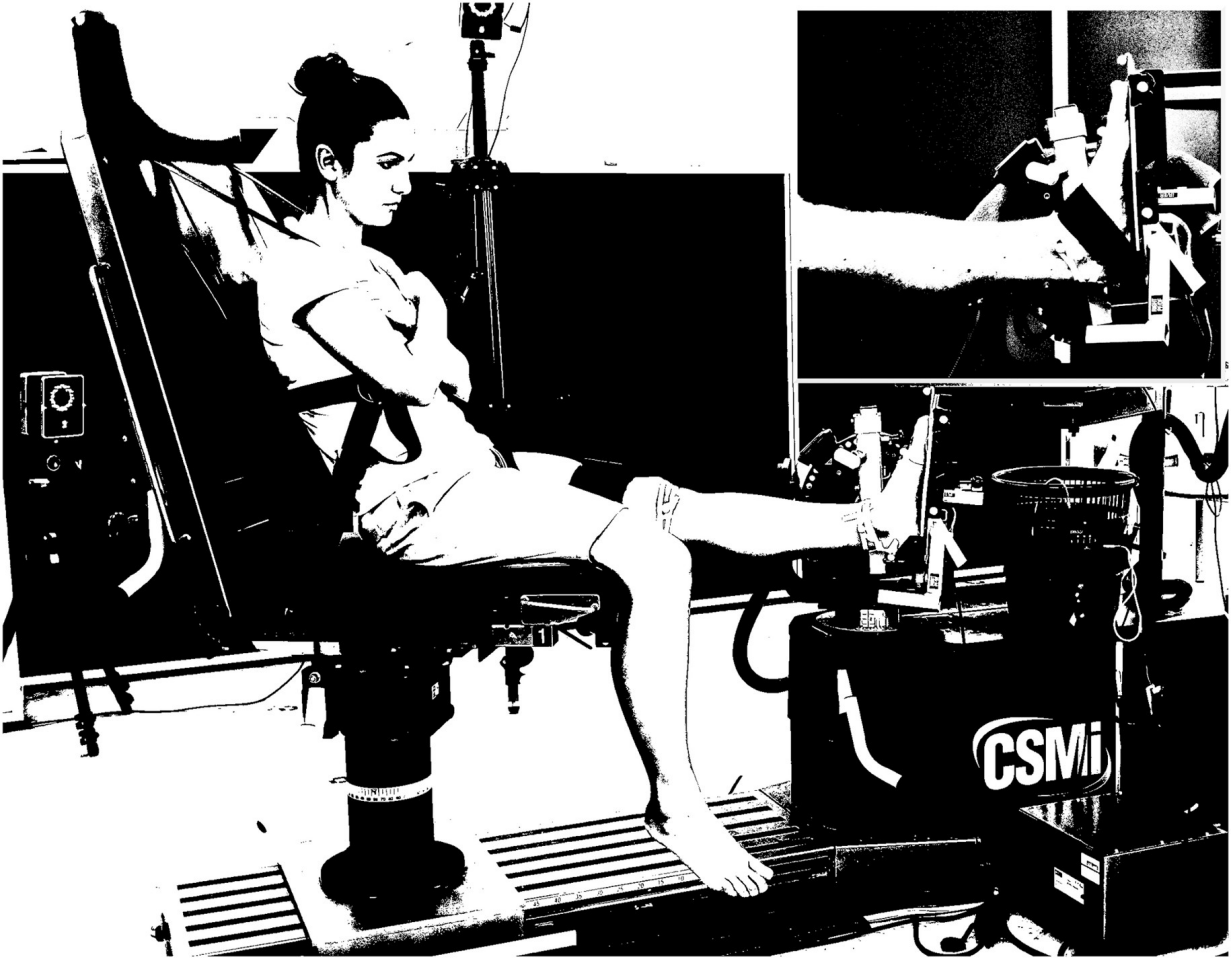
Experimental setup of the isometric plantarflexion contraction. The figure shows the participant seated on the dynamometer chair with their arms crossed and the upper body secured with belts. The left foot was placed on the dynamometer foot adapter (knee fully extended) and tested in two conditions (free-fixed) and four positions (0-9cm). The upper right figure depicting the secured foot with the inextensible strap. The individual pictured in Fig 1 has provided written informed consent (as outlined in PLOS consent form) to publish their image alongside the manuscript.

Fourteen healthy individuals (age 28.7 ± 6.9 yr., height 173.1 ± 7.3 cm, mass 69.1 ± 6.9 kg) volunteered to participate in the study. They were randomly recruited from the Centre for Sport Science and University Sports in Vienna where they regularly participate in physical activity. The participants did not have any major or recent musculoskeletal injury of the examined leg at the time of testing. All participants provided their written informed consent prior to participating in the study. Additionally, the individual pictured in Fig 1. has provided written informed consent (as outlined in PLOS consent form) to publish their image alongside the manuscript. The Ethical Committee of the University of Vienna (decision number 00422) approved the study.

The participants were asked to perform randomly maximal voluntary isometric contractions (MVC) in two different conditions, at four positions of the chair (Fig 1). Plantarflexions were performed with the foot either secured with inextensible straps (fixed) or free, while the dynamometer chair was anteriorly transported for 9 cm with 3 cm increments (0, 3, 6, 9 cm) to increase the pressure under the foot’s plantar surface [12]. The hip-knee-ankle joint angle configuration was similar for all participants (110-180-90°). We defined the straight hip and knee joint as 180° and the shank perpendicular to the foot as 90°. We placed the foot of the participants on the dynamometer footplate adapter (HUMAC NORM Model 770; CSMi, Stoughton, MA, USA) and we oriented the ankle joint rotational axis (defined as the midpoint of the line connecting both malleoli) to be coaxial to the dynamometer axis. During the test procedure, the participants were asked to hold their arms folded over their chest and they performed all plantarflexions unilaterally (left leg).

Prior to marker placement, the participants performed a warm-up session on a cycling ergometer (Kettler Ergometer PX1) for 8 minutes. Additionally, prior to testing, they also performed multiple submaximal and two maximal isometric plantarflexion contractions, for preconditioning purposes [35]. Following the warm-up, the participants were instructed to complete, at each position and condition, two ramp (3–4 s) maximal isometric voluntary (MVC) plantar flexion contractions and sustain them for ~2 s. During the contractions, the upper body and the left thigh were firmly secured with additional inextensible straps to prevent them from any involuntary motion. Between contractions, the thigh strap was loosened and one minute rest was given to prevent from muscle fatigue and any thixotropic effect [36,37]. The same investigator, using the same procedure, conducted all fixations and measurements.

For the positioning of the participants we used the same method described earlier [12]. Briefly, the neutral position (0cm), was first identified and then randomly the participants were moved to the next positions. In addition, at each position, the condition (fixed, free) was also randomly assigned. If a participant experienced pain or felt discomfort at the most anterior position (9cm) we moved them by one-centimeter increment to the next position (8 or 7 cm). Not all participants could achieve the last position (8.4±0.6cm) but for clarity purposes in this paper, we will refer to it as the position “9cm”.

All kinematics were captured by using the Vicon-MX-Motion-Capturing-System (Oxford, UK) with ten cameras operating at 120 Hz. For this purpose, reflective markers were placed on the following landmarks: the C7, trochanter major, the most prominent points of the lateral and medial femoral condyles (FC), lateral and medial malleolus (MM), the most prominent point of the tuber calcanei (TC), on the forefoot over the second metatarsal (FM) and on the top of the pressure insole. We also placed markers on the axis of the dynamometer, and two markers were placed on the footplate of the dynamometer to define the line of force application. The angle made by the FC, MM, and TC was defined as rearfoot angle, while the angle made by FC, TC and FM was defined as forefoot angle. We defined the knee and ankle joint center as the midpoint of the lines connecting both, the malleoli and femoral condyle. The kinematic data were low-pass filtered, using a fourth-order, zero phase-lag Butterworth filter with a cutoff frequency of 17 Hz [38].

The analog signal of the joint moment measured by the HUMAC isokinetic dynamometer was captured using the Vicon Nexus A/D card (16 bit) at 1200 Hz. The gravitational forces acting on the foot-dynamometer arm system were removed for all subjects prior to the voluntary contractions. We calculated the corrected joint moment through inverse dynamics by a method previously reported [20,23]. Briefly, we calculated the lever arm of the reaction force to the ankle joint, assuming a perpendicular force vector to the dynamometer footplate, by determining the point of force application under the foot using flexible pressure distribution insoles (Pedar-X; Novel GmbH, Germany; 100Hz) and used it as follows:

$$M_{corr}\;=\;Fd_{A}\;=\;M_{meas}\frac{d_{A}}{d_{B}}$$ (1)

where M_corr_ is the corrected joint moment, and F is the perpendicular force exerted on the dynamometer footplate at the point of force application. With d_A_ is the lever arm of the force (F) to the ankle joint, defined as the midpoint of both malleoli and d_B_ is the lever arm of the force (F) to the dynamometer axis. Finally, M_meas_ is the moment measured by the dynamometer device.

To synchronize all systems, we used a custom made trigger device (TTL, 0-5V) that was connected to both the Pedar-X and Vicon Nexus measuring systems [12]. All captured data (kinematic, pressure insole) were interpolated using cubic splines to achieve a common frequency (1200 Hz). The joint moment and pressure data were low-pass filtered with a fourth-order, zero phase-lag Butterworth filter using a cutoff frequency of 15 and 9 Hz, respectively.

We processed all data in Matlab 2019 (The MathWorks Inc., Natick, MA, USA) while the statistical analysis was performed using IBM SPSS Statistics 24 (IBM Corporation, NY, USA). We set the level of significance at 0.05, a priori for all analyses. To identify a possible effect of the independent variables (position and condition) to the examined dependent variables (moment, joint rotation), we conducted a two-way (within-within subject design) ANOVA with repeated measurements test. In case of a significant interaction effect, we examined the main effect and conducted a post-hoc test with Bonferroni correction, to identify the differences among the four positions (0, 3, 6 and 9 cm). Normal distribution was assessed with a Shapiro-Wilks-test and the effect size was determined by calculating partial eta squared (η^2^).

## Results

**Fig 2:**
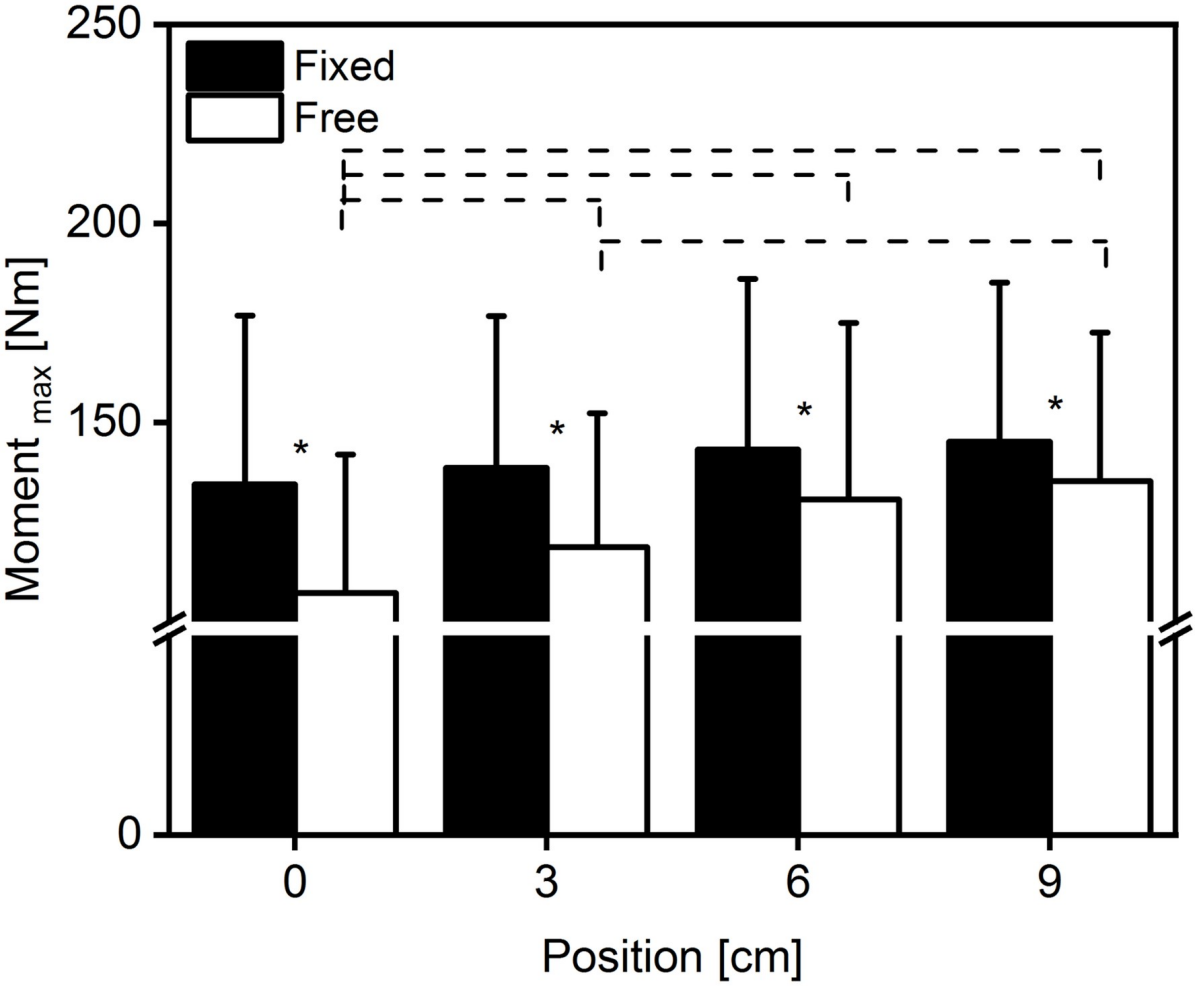
Average maximal plantarflexion moment at four positions and two conditions. Average (mean ± SD) maximum plantarflexion moment at four different positions (0–9 cm) and two different conditions (Fixed = filled bars, Free = empty bars). *: Indicates significant difference between conditions. Dashed line: Indicates significant difference between positions (n = 14).

We found a significant interaction of condition × position on the maximal plantarflexion moment F(3,39) = 7.953, p<0.001, η^2^ = 0.380, and a main effect for position F(3, 39) = 12.555 p<0.001 η^2^ = 0.491. We also found a significant main effect for condition F(1,13) = 21.341, p<0.001, η^2^ = 0.621 indicating that the fixation method affected the joint moment (Fig 2). The post hoc comparison of position revealed significant differences (p<0.05) only at free condition (Fig 2). Similarly, the post hoc comparison of condition revealed significant differences (p<0.05) between the fixed and free condition at all positions (Fig 2).

**Fig 3:**
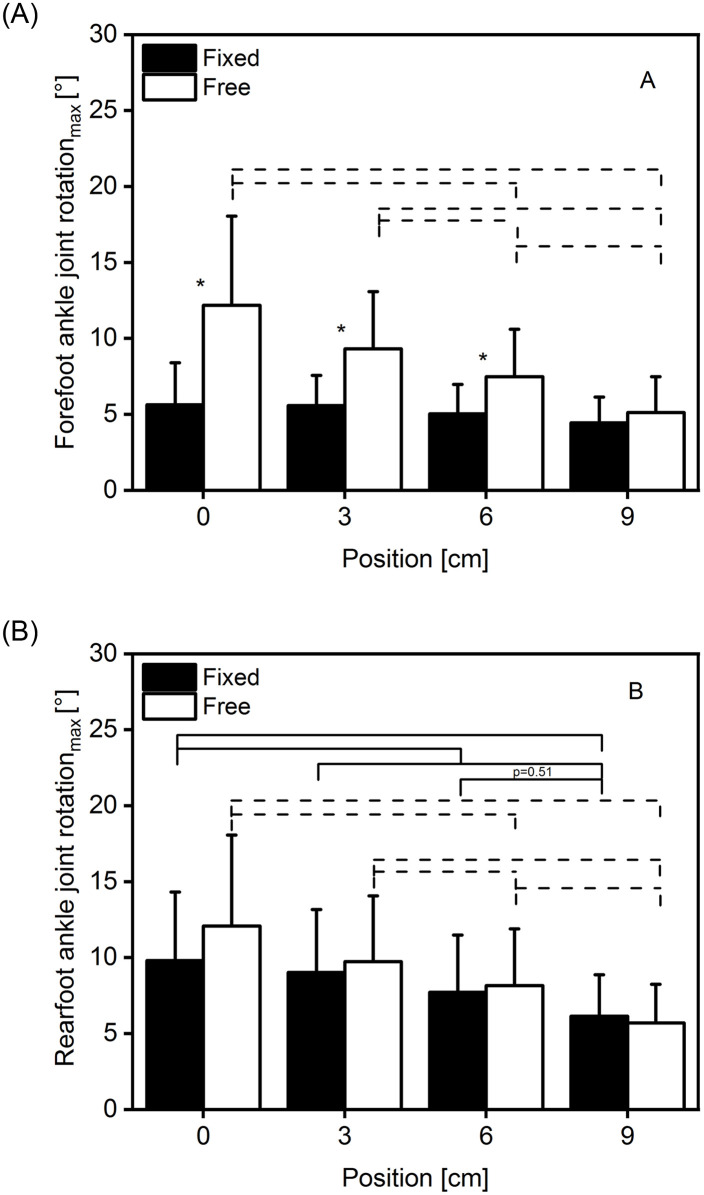
Maximal ankle joint rotation at four positions, two conditions and two measuring sites. Forefoot (A) and rearfoot (B) average (± SD) maximal ankle joint rotation during plantarflexion in fixed (filled bars) and free (empty bars) condition, at four (0–9 cm) positions (n = 14). Solid lines indicating significant difference (p<0.05) between positions at fixed condition, and dashed lines indicating significant difference between positions at free condition (p<0.05). *: Indicating significant difference (p<0.05) between conditions (n = 14).

We found a significant condition × position interaction on the forefoot joint rotation F(1.785, 23.202) = 21.999, p<0.001, η^2^ = 0.629, and a significant main effect for position F(1.787, 23.225) = 16.476 p<0.001 η^2^ = 0.559 (Fig 3A). Also, a significant main effect for condition could be found F(1,13) = 27.064 p<0.001 η^2^ = 0.676. Post hoc comparisons of position revealed significant differences (p<0.05) at free condition between the positions 0 to 6 and 9 cm, 3 to 6 and 9 cm, and between 6 and 9 cm (Fig 3A). Additionally, a significant difference between conditions was found at the positions 0, 3 and 6 cm (Fig 3A).

A significant interaction between condition x position on the rearfoot joint rotation could be found F(1.391,18.086) = 5.33, p = 0.024, η^2^ = 0.291 (Fig 3B). Furthermore, there was a significant main effect for position F(3,39) = 24.936 p<0.001 η^2^ = 0.657. We found no main effect for condition F(1,13) = 1.198 p>0.05 η^2^ = 0.132. Post hoc comparison revealed significant differences at fixed condition between the positions 0 to 6 and 9 cm, between the 3 and 9 cm and a tendency (p = 0.051) between the positions 6 and 9 cm (Fig 3B). Similarly, at free condition we found significant differences (p<0.05) between the positions 0 to 6 and 9 cm, between the 3 to 6 and 9 cm and between the 6 and 9 cm (Fig 3B). No significant difference was found between conditions at any position (Fig 3B).

**Fig 4:**
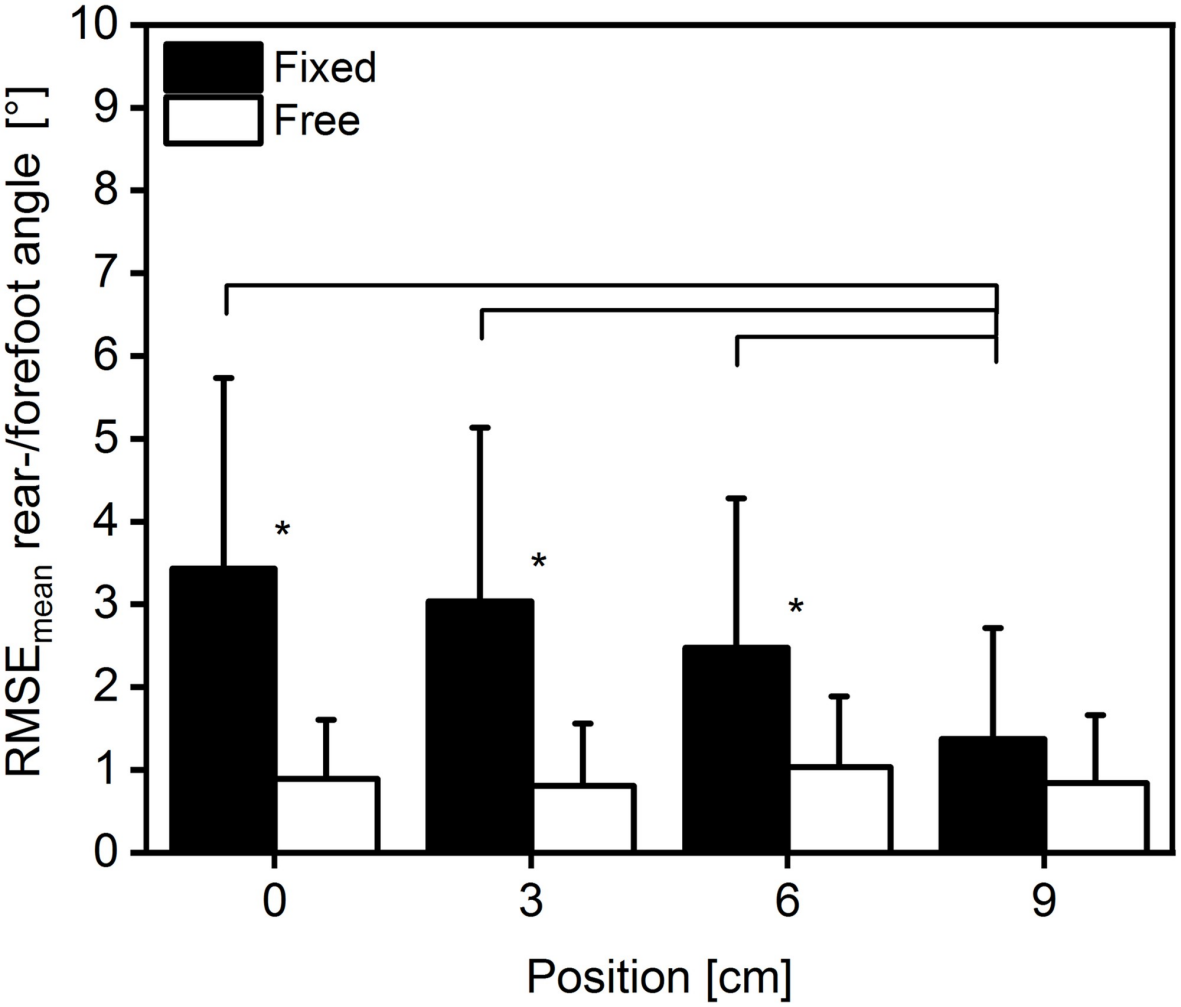
Root mean square error difference of joint rotation. Average (mean ± SD) root mean square error difference of the maximum ankle joint rotation, at four different positions (0–9 cm) and two different conditions (fixed = filled bars, free = empty bars). *: Indicates significant difference (p<0.05) between conditions. Solid line: Indicates significant difference (p<0.05) between positions (n = 14).

We calculated the root mean square error (RMSE) difference between the two measuring sites and found a significant interaction of condition × position on the RMSE_mean_ F(3,39) = 13.586, p<0.001, η^2^ = 0.511. There was a significant main effect for position F(3, 39) = 8.575 p<0.01 η^2^ = 0.397 and for condition F(1,13) = 21.623 p<0.01, η^2^ = 0.625. The post hoc comparison for position showed significant differences (p<0.05) between positions 0–6 with 9 cm (Fig 4) only at the fixed condition. Additionally, the post hoc comparison for condition revealed significant differences between the positions 0, 3, and 6 cm (Fig 4).

No significant differences between positions could be found for the Ankle-Joint-Angle at rest in the rearfoot and forefoot measuring site, the Knee-Joint-Angle at rest and at maximum, and the Hip-Joint-Angle at rest (Table 1) indicating similar initial test-conditions. The Hip-Joint-Angle at max showed, at position 0 cm, significant differences from the positions 3–9 cm at both conditions (Table 1). Additionally, the foot pressure increased significantly from 0 to 9 cm (Table 1).

**Table 1:** Average (mean ±SD) values of the foot pressure at rest, forefoot and rearfoot ankle joint angle at rest, knee joint angle at rest and maximum, hip joint angle at rest and maximum, at the four positions (0–9 cm) and two conditions (fixed, free).

Parameter/position	Fixed				Free			
	0cm	3cm	6cm	9cm	0cm	3cm	6cm	9cm
Foot Pressure _rest_ [kPa]	79.4 ± 39.5^6−9^	116.8 ± 37.5^6−9^	183.2 ± 59.2^9^	282.0 ± 91.2	65.1 ± 22.6^3−6–9^	112.8 ± 33.0^6−9^	182.6 ± 67.8^9^	285.2 ± 98.2
Ankle Joint _rest_ forefoot [°]	90.3 ± 0.9	89.7 ± 0.8	89.4 ± 1.3	89.7 ± 1.5	90.9 ± 0.9	90.1 ± 1.1	89.5 ± 1.4	89.7 ± 1.4
Ankle Joint _rest_ rearfoot [°]	89.5 ± 2.6	90.2 ± 2.5	90.4 ± 2.3	91.0 ± 2.6	91.3 ± 1.3	91.5 ± 1.3	91.5 ± 1.3	92.6 ± 1.5
Knee Joint _rest_ [°]	180.0 ± 0.9	180.1 ± 0.9	180.4 ± 1.3	180.3 ± 1.5	180.0 ± 1.5	179.9 ± 1.5	179.2 ± 2.3	179.1 ± 2.4
Knee Joint _max_ [°]	180.6 ± 2.1	179.3 ± 2.8	180.6 ± 1.6	179.9 ± 2.7	180.4 ± 2.3	179.4 ± 2.9	180.6 ± 1.5	179.9 ± 2.8
Hip Joint _rest_ [°]	124.3 ± 7.2	122.1 ± 6.7	121.3 ± 7.1	121.1 ± 7.4	124.3 ± 6.9	122.3 ± 7.4	121.6 ± 6.4	120.4 ± 7.6
Hip Joint _max_ [°]	125.5 ± 6.8^3−6–9^	120.8 ± 7.0	119.8 ± 5.4	119.4 ± 5.8	123.8 ± 7.8^3−6–9^	119.4 ± 6.8	119.9 ± 6.2	119.1 ± 6.8

Superscript numbers (3-6-9) indicating significant differences (p<0.05) between positions at the same condition (n = 14).

## Discussion

The first aim of this study was to examine the foot straps effectiveness on the generated moment. We hypothesized that foot strap and increased foot pressure would result to similar torque development. We could not confirm the first hypothesis, since we found a significant higher plantarflexion moment at all positions for the fixed (straps) compared to the free condition. The second aim of the study was to compare the joint rotation in respect to the measuring site (forefoot–rearfoot and condition) and we hypothesized different measuring outcomes. We could confirm our second hypothesis since the foot fixation affected the amount of ankle joint rotation in dependence of the measuring site.

In order to reduce the dynamometer-subject elasticity during plantarflexion contractions, researchers implemented different methods [12,25,26]. In an previous attempt [12], with the foot unrestrained, the maximum plantarflexion moment increased by >32% by merely increasing the foot pressure on the dynamometer-foot adapter. In the present study, the percentage increase of moment in the free condition was ~26% at the last position (9 cm), and this is practically comparable with the previous reported values [12]. The small difference can be attributed to the different foot pressure developed at the first position (0 cm) of both studies (65.1 and 71.7 kPa, present and previous respectively). The different initial conditions could influence the foot pressure at the last position (282 and 339 kPa at 9 cm, present and previous respectively) and, as a consequence, the moment development. Interestingly, Cannavan and colleagues [26], in their pilot study, did not find any significant difference in the force levels between their novel and traditional set-up, which is in accordance to our present results for the fixed condition over all positions (Fig 2). In this study, we could not find a significant difference in the exerted moment at the fixed condition between any positions (Fig 2), indicating that with a proper foot fixation, the maximum plantarflexion moment can be achieved regardless of the position. Additionally, we compared (paired t-test) separately the exerted moment in free condition at the last position (9 cm) with the fixed condition at positions 0–6 cm and did not find any significant difference (p>0.05). This result suggests that, in order to achieve a maximal plantarflexion moment without proper fixation (straps), it is necessary to maximally reduce the compliance of the dynamometer-subject system, for example by forward positioning of the dynamometer chair.

**Fig 5:**
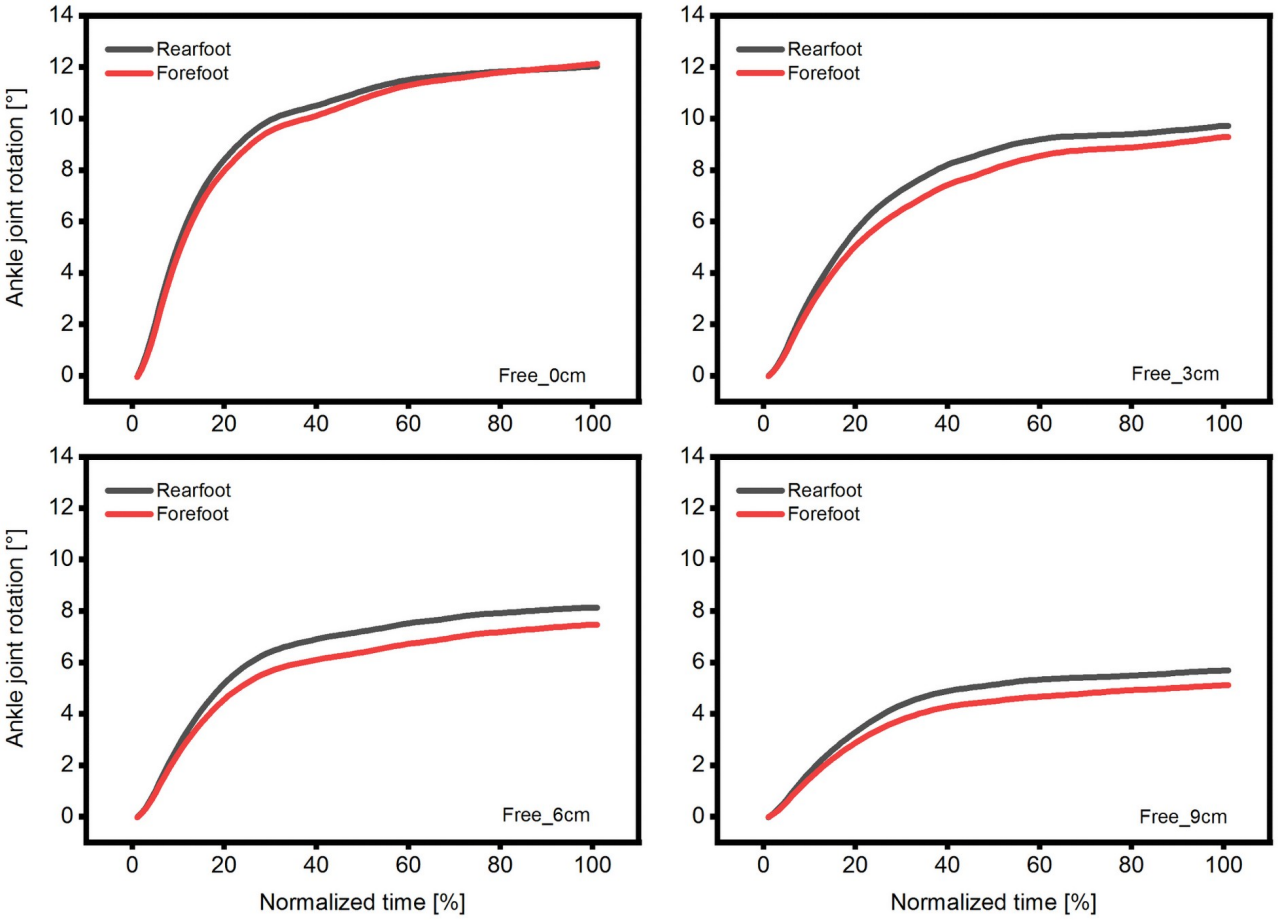
Time normalized ankle joint rotation at four different positions in free condition. Time normalized average ankle joint angle measured in rearfoot and forefoot measuring sites at four different positions (0–9 cm) and in free condition. For clarity, the standard deviation is not presented (n = 14).

**Fig 6:**
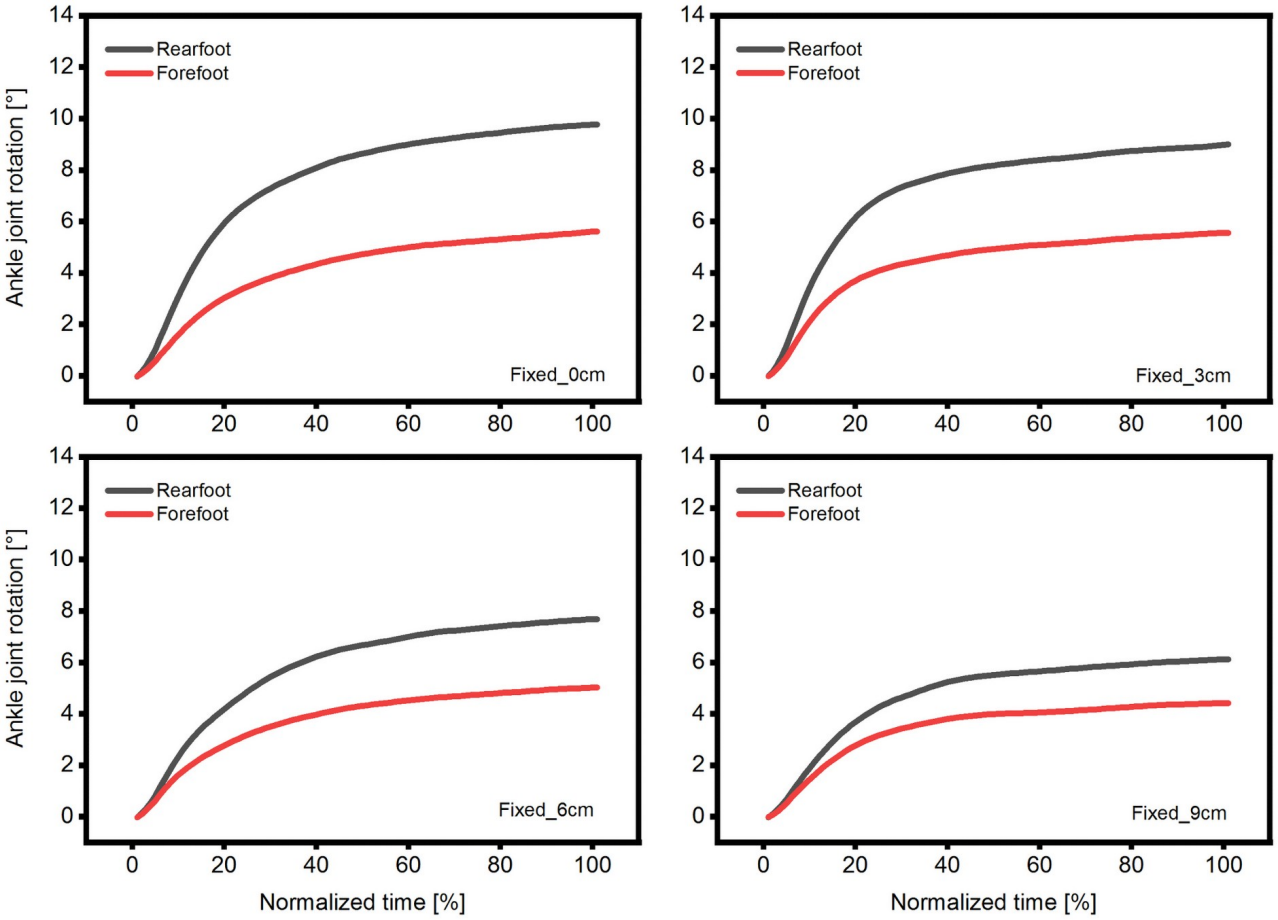
Time normalized ankle joint rotation at four different positions in fixed condition. Time normalized average ankle joint angle measured in rearfoot and forefoot measuring sites at four different positions (0–9 cm) and in fixed condition. For clarity, the standard deviation is not presented (n = 14).

During the plantarflexion effort, the ankle joint rotated irrespective of the position, fixation or measuring method (Figs 3A and 3B and 5 & 6). The lowest values were recorded when measuring the forefoot in fixed condition (averaged collapsed data ~5°; Fig 3A) and the highest values when measuring the rearfoot in the free condition (averaged collapsed data ~9°; Fig 3B). The reduced dynamometer-subject compliance affected the max joint rotation, and as a result, we found a reduction of 21 and 58% in the forefoot-fixed and free method (Fig 3A) and 37 and 53% in the rearfoot- fixed and free method (Fig 3B), respectively. We reported similar results previously during unrestrained plantarflexions with a joint rotation reduction of 54% in the most anterior position [12], and hence we can confirm the previous findings. Also other researchers [25,26] reported a reduction in the ankle joint rotation by means of reducing the dynamometer-subject compliance. Those findings confirm the aforementioned method when the goal of the study is to decrease the ankle joint rotation. Nonetheless, the two different measuring methods (forefoot, rearfoot) showed different kinematic results in dependence of the fixation method (Figs 3A and 3B and 4). We calculated the root mean square error difference of the forefoot-rearfoot method and found a significant higher difference in the fixed condition that was reduced by decreased dynamometer subject compliance (Fig 4). Since many researchers use inextensible straps [4,5,14], the choice of measuring site (forefoot or rearfoot) could influence the assessment of the joint rotation (Fig 3A & 3B). Additionally, it appears that the ankle joint rotation assessed in the rearfoot is not affected by the fixation method, since we could not find any significant difference between the two fixation methods at any position (Figs 3B and 5). Furthermore, this could indicate that the values of the rearfoot measuring site are more robust to condition (fixed or free) or to subject positioning. We additionally compared (paired t-test) the maximal rearfoot with the maximal forefoot joint rotation in the free condition at the respective positions, and found no significant difference (p>0.05) between the respective values. This could further indicate that when no fixation method is used, the measuring site (forefoot or rearfoot) is not affecting the measuring outcome. This finding could help future researchers who implement complex foot models [39] during plantar flexions and do not want to be restricted by the use of the inextensible straps.

Conversely, when the scope of the study is the minimization of the ankle-joint movement on an isokinetic dynamometer, the implementation of inextensible straps is necessary (Fig 3A). It can be argued if different straps from different manufacturers have the same effect on the joint rotation, but since we did not examine that aspect, we can point out that only with a proper fixation, a reduction of the ankle joint rotation can be achieved. Additionally, minimal ankle rotation was observed when the fixed-forefoot measuring site was chosen, indicating that not only the fixation method, but the measuring site, as well, is important for the reduction of the ankle joint rotation (Fig 3A). The mechanism responsible for that behavior could be attributed to the multi-segment structure of the foot [31]. The straps are generally placed over the dorsal venous arch of the midfoot, which is expected to compress and reduce the movement of the talocalcaneonavicular, calcaneocuboid and tarsometatarsal joint. Owing to that, it appears that all joints, included in the forefoot marker-setup, to function as one unit. This is apparent in the Fig 3A where the mean joint rotation for the forefoot measuring site was approximately 5° over all positions in the fixed condition and without any significant difference. Moreover, the same marker setting without fixation (free condition) enabled the joint to rotate approximately 8.5° (average over all positions) with significant differences between positions (Fig 3A).

In the literature we find studies [1,14,24] that assessed the ankle joint rotation in order to implement corrections of the tendon displacement attributed to the inevitable joint movement [14,20,24]. Therefore, the measuring site (forefoot, rearfoot) could play an important role in the over- or underestimation of the joint rotation. For example, in the present study, the absolute angle difference of the two different measuring sites (forefoot, rearfoot) at the first position (0 cm) and the fixed condition was ~4° (Fig 3A & 3B), which could result in an overestimation of the tendon displacement by 2.8 mm, assuming an average displacement ratio of 0.7 mm/° [24]. Additionally, assuming a tendon length of 150 mm, this overestimation could result in a 1.8% strain increase, which can be considered as substantial. Unfortunately, with the present results, we cannot suggest an appropriate measuring site that would measure the “real” ankle joint rotation. It appears that the free condition (no straps) provides similar angular results independent of position or measuring site (Fig 4) and therefore could probably be used to assess the joint rotation, but with a tradeoff in the exerted moment.

## Limitation

Although the hip joint angle at rest did not show any significant difference, at maximum we found a significant difference at position 0 to 3–6 and 9 cm at both conditions (Table 1). Additionally we tested (paired t-test, normal distributed data) the hip and knee joint angles between Rest and Max and found significant (p < .05) reduction of the hip joint angle (~2°) at the position 3cm (fixed condition) and 9cm (fixed and free condition). No difference was found in the knee joint angle at all positions and conditions. The greater hip flexion in the positions 3–9 cm and between the rest and max can be attributed to the deformation of the cushioning back pad produced by the increased pressure and the increased force development. Nonetheless, the hip flexion (~4.5°) indicates that the contribution of the back and trunk joints is unlikely to influence the plantarflexion moment [40] and thus affect the outcome of this study.

It is generally accepted that a reliability analysis can improve the scientific findings of a study. The current set-up was not tested for reliability and that could possibly constitute a limitation of the present study.

## Conclusion

The results of this study indicate that the fixation method affects the generated plantarflexion moment and the extent of joint rotation. Additionally, when no fixation is applied, the choice of ankle joint measuring site (forefoot, rearfoot) does not affect the measured joint rotation. Therefore, this method could be implemented in trials where the foot should be monitored under unconstrained conditions. In trials where the maximum generated plantarflexion moment and the minimum ankle joint rotation is required, the implementation of foot straps combined with forefoot measuring site is necessary.

## Supporting information

S1 Data Includes all related data. (XLSX)
